# Factors associated with trunk skeletal muscle thickness and echo intensity in young Japanese men and women

**DOI:** 10.1371/journal.pone.0312523

**Published:** 2025-01-06

**Authors:** Funa Kitagawa, Hiroshi Akima, Noriko Ishiguro-Tanaka

**Affiliations:** 1 Graduate School of Education & Human Development, Nagoya University, Nagoya, Aichi, Japan; 2 Japan Society for the Promotion of Science (JSPS), Chiyoda, Tokyo, Japan; 3 Research Center of Health, Physical Fitness & Sports, Nagoya University, Nagoya, Aichi, Japan; Ritsumeikan University, JAPAN

## Abstract

The present study examined factors associated with trunk skeletal muscle thickness (MT, an index for the amount of skeletal muscle) and echo intensity (EI, an index for the content of non-contractile tissue, such as intramuscular adipose tissue) in young Japanese men and women in consideration of habitual dietary intake. Healthy men (n = 26) and women (n = 24) aged 20 to 26 were enrolled. Trunk MT and EI were evaluated using ultrasound imaging at the height of the 3rd lumbar vertebra. In addition to morphological variables, brachial-ankle pulse wave velocity (baPWV) and blood properties (e.g., triglycerides, total cholesterol, and fasting blood glucose) were measured. Habitual dietary intake was also evaluated by a self-administered diet history questionnaire. The results obtained for young men revealed significant correlations between trunk MT/body mass^1/3^ and the percentages of energy from polyunsaturated fatty acids (*r*_*s*_ = 0.476, p <0.05) and carbohydrates (*r*_*s*_ = -0.402, p <0.05). Trunk EI significantly and positively correlated with the percentage of energy from saturated fatty acids (*r*_*s*_ = 0.397, p <0.05). In young women, trunk EI showed a significant and positive correlation with baPWV (*r*_*s*_ = 0.504, p <0.05). These results suggest that the effects of habitual dietary intake on trunk skeletal muscle differ between young men and women.

## Introduction

The atrophy of skeletal muscle (SM) and content of non-contractile tissue, such as intramuscular adipose tissue, significantly correlate with cardiometabolic risks (e.g., [1, 2]) and physical impairments (e.g., [3, 4]). The atrophy of SM and content of intramuscular adipose tissue have been reported in old and young individuals [5]. Previous studies indicated that SM atrophy occurs earlier in the trunk than in the extremities [6, 7]. In the thigh, the content of intramuscular adipose tissue has been shown to significantly and negatively correlate with the amount of SM [8]. These findings indicate that intramuscular adipose tissue accumulates earlier in the trunk than in the extremities. In addition, Ido et al. [9] reported that the abdominal skeletal muscle thickness (MT) was significantly associated with metabolic risk scores, while such relationships were not identified in other MT including that in the thigh. On the other hand, the content of intramuscular adipose tissue in the trunk was more strongly associated with visceral adipose tissue and waist circumference compared with that in the thigh [10]. Therefore, the identification of factors associated with the amount of SM and content of intramuscular adipose tissue in the trunk is important for reducing cardiometabolic risk factors and preventing physical impairments.

Dietary intake generally regulates the dynamic balance between muscle protein synthesis and breakdown [11] Previous studies on healthy older adults suggested that the amount of total daily protein intake promoted muscle protein synthesis and maintained the amount of SM and physical performance, such as balance ability, walking speed, and muscle strength [12]. Weight-loss intervention studies indicated that a high-protein diet maintained the amount of SM in overweight or obese older adults (e.g., [13]). However, it currently remains unclear whether habitual dietary intake, particularly micronutrients, affects the amount of SM and content of intramuscular adipose tissue in the trunk. To the best of our knowledge, only Kitagawa et al. [14] has examined the relationship between habitual micronutrient intake and the content of intramuscular adipose tissue in the trunk. The reported a significant correlation in older men, but not in younger men. However, they did not investigate the amount of trunk SM or women. The body composition of men and women differs due to the effects of sex hormones. Therefore, sex differences in the factors associated with the amount of SM and content of intramuscular adipose tissue in the trunk in young adulthood need to be examined.

The gold standard for evaluating the amount of SM and content of intramuscular adipose tissue is imaging techniques, such as magnetic resonance imaging and computed tomography. However, these methods are expensive and time-consuming to analyze. Furthermore, since computed tomography involves x-ray exposure, it is prohibited for pregnant women. On the other hand, ultrasound imaging is a low-cost, safe, and widely-available technique. Ultrasound images provide information on MT, an index of the amount of SM [15], and echo intensity (EI), an index of the content of non-contractile tissue, mainly intramuscular adipose tissue [16, 17]. Therefore, the present study examined factors associated with trunk MT and EI in young Japanese men and women in consideration of dietary intake. We hypothesized that habitual dietary intake significantly correlates with trunk MT and EI, and factors associated with trunk MT and EI may differ between men and women.

## Materials and methods

### Participants

We collected data from 50 healthy Japanese men (n = 26, body mass index; BMI: 21.0 ± 1.9 kg/m^2^) and women (n = 24, BMI: 19.6 ± 1.9 kg/m^2^) aged 20 to 26 between February 2021 and February 2022. None of the participants had an exercise habit [18]. We used leaflets and a website to recruit participants. Before the initiation of data collection, we orally explained the procedure, purposes, risks, and benefits associated with this study to participants and obtained their written consent. All data collection was conducted at our university. We calculated the sample size for correlations using G*Power software, namely, with two tails, an effect size of 0.4, a desired statistical power level of 0.8, and a probability level of 0.05. As a result, the minimum sample size was 19 in each group. Accounting for dropouts and increasing the validity of our results, we recruited more subjects than the minimum sample size. The present study was approved by the Ethics Committee of our university (approval No. 20–11) and was conducted according to the principles of the Declaration of Helsinki.

### Morphological measurements

Height was assessed using a standard physician’s scale. Body mass (BM) was measured by an electric scale. Waist circumference measurements were performed at the level of the umbilicus.

### Ultrasound imaging

A portable B-mode ultrasound device (Logiq e Pro: GE Health Care Co., Ltd., USA) was applied to obtain cross-sectional images from the abdominal, lateral, and lower back on the right side at the height of the third lumbar vertebra. The height of the third lumbar vertebra was reported as the best reference site for assessing total tissue volume of SM, visceral adipose tissue, and subcutaneous adipose tissue [19, 20]. First, the height of the intervertebral disc between the fourth and fifth lumbar vertebrae was determined from Jacobi lines. Second, the height of the third lumbar vertebrae was determined by ultrasound imaging with the participant lying prone on a bed [21]. The same ultrasound settings (frequency: 10 Hz, gain: 45 dB) were used for each scan. Participants lay in the supine position to acquire abdominal images, the lateral decubitus position for lateral images, and the prone position for lower back images. During the acquisition of abdominal and lateral images, subjects were instructed to hold their breath for several seconds after inhalation. After a 10 minute-rest to mitigate the influence of fluid-shift [21], three ultrasound images were acquired from each region. All ultrasound images were analyzed by ImageJ (version 1.47v, National Institutes of Health, USA). MT and EI were measured for three trunk SM groups, namely, the rectus abdominis, external oblique abdominal, and lumbar multifidus muscles. MT was defined as the distance from subcutaneous adipose tissue—SM interfaces to the SM—abdominal cavity, SM—SM interfaces, or SM—bone interfaces [15, 21]. To measure EI, a rectangular region of interest was set as large as possible within each SM and the mean intensity within it was assessed using a standard histogram function on a scale of 0 to 255 (black = 0, white = 255) [22]. All three images taken from each region were analyzed to obtain MT and EI and average values were used in analyses. All measurements were performed by one experienced expert (N.T.). Test-retest reliability (ICC_1,1_) was 0.954 for MT and 0.926 for EI.

### Arterial stiffness

Brachial-ankle pulse wave velocity (baPWV) was measured as an index of arterial stiffness (FORM-5, Fukuda Colin, Tokyo, Japan). Measurements were performed after an at least 10-minute rest in the supine position. Blood pressure cuffs were wrapped on both arms and ankles. This procedure has been described in detail in previous studies (e.g., [23]).

### Blood properties

After an overnight fast ≥12 h, blood was sampled from the antecubital vein and sent to a commercial laboratory (LSI Medience, Tokyo, Japan) for analysis. Triglycerides, total cholesterol, high-density lipoprotein-cholesterol (HDL-cholesterol), fasting blood glucose, and insulin were analyzed. We calculated indices of insulin resistance, such as the homeostasis model of assessment-insulin resistance (HOMA-IR) and homeostasis model of assessment-and quantitative insulin sensitivity check index (QUICKI). The index of insulin secretion was also evaluated with the homeostatic model assessment of beta cell function (HOMA-β). These indices were calculated using the following equations:

HOMA-IR = fasting blood glucose (mg/dL) × fasting insulin (μIU/mL) / 405

QUICKI = 1 / [log fasting plasma insulin (lU/mL) + log fasting plasma glucose

(mg/dL)]

HOMA-β = fasting insulin (μIU/mL) × 360 / [fasting blood glucose (mg/dL) - 63]

### Habitual dietary intake

A brief-type self-administered diet history questionnaire (BDHQ) for Japanese adults was used to assess habitual dietary intake in the previous month [24]. Details on the structure and calculation of habitual dietary intake by BDHQ have been described elsewhere [25, 26]. We focused on total energy intake and the percentages of energy from protein, fat, carbohydrate, saturated fatty acids (SFA), monounsaturated fatty acids, and polyunsaturated fatty acids (PUFA). The nutrient adequacy score (NAS) was evaluated by the intake of 10 nutrients (dietary fiber, vitamin B₆, vitamin D, vitamin K, folic acid, pantothenic acid, potassium, calcium, magnesium, and manganese) [14]. A low NAS indicates lower intakes of the 10 nutrients than the dietary reference intakes recommended in Japan [27].

### Physical activity

The global physical activity questionnaire (GPAQ) was used to assess daily physical activity and total energy expenditure. The GPAQ consists of questions to determine physical activity according to behavioral domains such as work, transportation, leisure and sedentary activity. Detailed information on the GPAQ is described elsewhere [28].

### Statistical analysis

Since many data showed non-normality distributed by the Shapiro-Wilk test, all data are shown as medians and 25^th^ to 75^th^ percentile values (Table 1). The Mann-Whitney U test was used to assess the significance of differences between young men and women. In general, there is the obvious sex-related difference in body size. To adjust for these differences, MT was corrected in the same way as by Kanehisa et al. [29]. Namely, in addition to the absolute value of MT, MT/BM^1/3^ was also calculated. This index is based on the theory that the diameter of various mammalian organs is a function of BM^1/3^ [30]. With this adjustment, we can compare the factors associated with trunk tissue composition equally between men and women. Indeed, there was a significant correlation between the absolute value of MT and MT/BM^1/3^ for both sexes (r_s_ = 0.899 for men and 0.918 for women, p<0.01). However, the items that were significantly related with MT and MT/BM^1/3^ were different. The relationships between trunk MT/BM^1/3^ or trunk EI and other parameters were analyzed using Spearman’s rank correlation coefficients. All statistical analyses were set at a significance level of 5%. All data were processed using IBM SPSS Statistics 20 (IBM Japan, Tokyo, Japan).

**Table 1:** Characteristics of participants.

	Men (n = 26)				Women (n = 24)					
Items	Median	25%	-	75%	Median	25%	-	75%	p	
**Anthropometry**										
Age (years)	22.0	20.0	-	23.0	22.0	21.0	-	23.0	0.503	
Height (cm)	170.7	166.0	-	176.6	159.7	155.7	-	164.2	<0.001	**
BM (kg)	61.9	56.5	-	65.8	50.1	46.6	-	54.0	<0.001	**
BMI (kg/m^2^)	20.9	19.8	-	22.0	19.6	18.1	-	20.8	0.012	*
Waist circumference (cm)	74.0	72.6	-	78.1	70.0	66.7	-	72.5	0.003	**
Trunk MT (mm)	16.5	15.4	-	17.5	13.8	12.7	-	14.9	<0.001	**
Trunk MT/BM^1/3^ (mm/kg^1/3^)	4.2	3.9	-	4.5	3.6	3.5	-	4.0	<0.001	**
Trunk EI (a.u.)	51.4	42.5	-	58.5	55.8	47.0	-	64.6	0.229	
**Arterial stiffness**										
baPWV (cm/s)	1080.3	995.1	-	1141.4	994.0	886.1	-	1066.6	0.010	*
**Blood properties**										
Triglycerides (mg/dL)	67.0	52.5	-	85.5	56.0	40.5	-	74.3	0.174	
Total cholesterol (mg/dL)	167.5	147.3	-	187.8	190.0	168.5	-	205.3	0.065	
HDL-cholesterol (mg/dL)	57.0	51.3	-	60.8	73.0	63.8	-	79.3	<0.001	**
Glucose (mg/dl)	79.5	77.3	-	85.0	81.0	76.8	-	84.8	0.969	
Insulin (μU/mL)	4.2	2.8	-	5.2	4.5	3.7	-	6.3	0.346	
HOMA-IR	0.8	0.5	-	1.1	0.9	0.7	-	1.3	0.351	
QUICKI	0.4	0.4	-	0.4	0.4	0.4	-	0.4	0.297	
HOMA-β	83.4	58.1	-	108.0	108.0	76.5	-	128.3	0.174	
**Dietary intakes**										
Energy intake (kcal)	1996	1664	-	2763	1540	1186	-	1768	0.003	**
Protein (%)	14.8	12.8	-	15.6	14.1	13.1	-	15.6	0.923	
Fat (%)	29.9	23.0	-	32.8	31.9	27.7	-	35.7	0.156	
Carbohydrates (%)	55.0	52.8	-	65.3	53.2	48.8	-	59.6	0.236	
SFA (%)	7.8	6.5	-	10.0	8.7	7.5	-	10.2	0.214	
MUFA (%)	11.0	8.5	-	11.9	11.4	9.8	-	13.5	0.351	
PUFA (%)	6.8	5.7	-	7.5	7.2	6.4	-	9.0	0.174	
NAS	6.0	4.0	-	8.0	6.5	4.0	-	8.0	0.992	
**Physical activity**										
Total energy expenditure (Kcal)	1735.7	1638.8	-	1978.8	1452.0	1305.8	-	1527.2	<0.001	**

BM: body mass, BMI: body mass index, MT: muscle thickness, EI: echo intensity, baPWV: brachial-ankle pulse wave velocity, HDL-cholesterol: high-density lipoprotein cholesterol, HOMA-IR: homeostatic model assessment for insulin resistance, QUICKI: quantitative insulin sensitivity check index, HOMA-β: homeostatic model assessment of beta cell function, SFA: saturated fatty acids, MUFA: monounsaturated fatty acids, PUFA: polyunsaturated fatty acids, NAS: nutritional adequacy score

** p <0.01

* p <0.05

## Results

Table 1 shows the characteristics of participants. Height, BM, waist circumference, trunk MT/BM^1/3^, baPWV, and energy intake were significantly higher in men than in women. In contrast, total cholesterol and HDL-cholesterol were significantly lower in men than in women. No significant group differences were observed in the other items tested. Among women, 33.3% were classified as underweight (BMI <18.5 kg/m^2^), while the corresponding value in men was 7.7%. In terms of nutritional intake, some participants did not meet the tentative dietary goal for preventing lifestyle-related diseases in the dietary reference intakes recommended in Japan (2020) [27]: 34.6% of men and 29.2% of women for protein (<50% or >65% of energy), 53.9% of men and 70.9% of women for fat (<13% or >20% of energy), and 8% of women and 5% of men for carbohydrates (<50% or >65% of energy). In addition, the percentage of participants with a higher percentage of energy from SFA (>7% of energy) than the tentative dietary goal in the dietary reference intake for Japanese (2020) [27] was 69.2% in men and 83.3% in women.

Correlations between trunk MT/BM^1/3^ or EI and other parameters are shown in Table 2. In men, trunk MT/BM^1/3^ significantly correlated with the percentages of energy from PUFA (*r*_*s*_ = 0.476, p <0.05) and carbohydrates (*r*_*s*_ = -0.402, p <0.05). In women, trunk MT/BM^1/3^ was significant correlations with fasting blood glucose, HOMA-IR, and QUICKI (*r*_*s*_ = 0.443, 0.407, and -0.406, p <0.05). However, these correlations were not observed following the exclusion of data from 1 participant (BMI: 17.1 kg/m^2^) whose fasting blood glucose and insulin were below the standard values recommended in Japan (fasting blood glucose ≤99 mg/dL [31], fasting blood insulin from 2.7 to 10.4 μU/mL [32]) (*r*_*s*_ = 0.396 for fasting blood glucose, 0.348 for HOMA-IR, and -0.347 for QUICKI, n.s.). Trunk EI significantly and positively correlated with the percentage of energy from SFA in men (*r*_*s*_ = 0.397, p <0.05). In women, a significant and positive correlation was observed between trunk EI and baPWV (*r*_*s*_ = 0.504, p <0.05). Other variables tested did not significantly correlate with trunk MT/BM^1/3^ or EI (*r*_*s*_ = -0.383 to 0.365, n.s.).

**Table 2:** Correlation coefficients (r_s_) with trunk MT/BM^1/3^ and EI variables.

	Trunk MT/BM^1/3^					Trunk EI					
	Men			Women		Men			Women		
Items	*r*_*s*_	*p*		*r*_*s*_	*p*	*r*_*s*_	*p*		*r*_*s*_	*p *	
Trunk EI (a.u.)	0.261	0.198		-0.181	0.398	-	-		-	-	
Age (years)	0.145	0.480		0.030	0.888	0.196	0.338		-0.035	0.870	
Height (cm)	-0.140	0.496		-0.116	0.589	-0.241	0.235		0.042	0.846	
BM (kg)	-0.079	0.703		0.155	0.469	-0.383	0.053		-0.129	0.547	
BMI (kg/m^2^)	0.145	0.479		0.230	0.279	-0.345	0.084		-0.168	0.433	
Waist circumference (cm)	0.121	0.557		0.047	0.826	-0.087	0.672		-0.066	0.759	
**Arterial stiffness**											
baPWV (cm/s)	-0.005	0.980		0.134	0.533	-0.028	0.893		0.504	0.012	*
**Blood properties**											
Triglycerides (mg/dL)	-0.063	0.758		0.131	0.541	0.057	0.783		-0.033	0.880	
Total cholesterol (mg/dL)	0.100	0.627		0.110	0.610	0.147	0.475		-0.221	0.298	
HDL-cholesterol (mg/dL)	-0.022	0.915		-0.257	0.225	0.171	0.405		-0.223	0.294	
Glucose (mg/dl)	-0.130	0.527		0.443	0.030	-0.062	0.762		-0.166	0.439	
Insulin (μU/mL)	-0.195	0.340		0.369	0.076	-0.043	0.833		-0.157	0.465	
HOMA-IR	-0.221	0.278		0.407	0.048	-0.050	0.807		-0.142	0.509	
QUICKI	0.201	0.325		-0.406	0.049	0.017	0.934		0.140	0.513	
HOMA-β	-0.130	0.526		-0.086	0.691	0.024	0.909		0.072	0.739	
**Dietary intakes**											
Energy intake (kcal)	-0.182	0.373		0.099	0.645	-0.204	0.317		0.106	0.622	
Protein (%)	0.309	0.124		-0.139	0.517	0.162	0.430		-0.070	0.747	
Fat (%)	0.365	0.066		-0.202	0.344	0.296	0.141		0.008	0.971	
Carbohydrates (%)	-0.402	0.042	*	0.220	0.302	-0.325	0.105		0.043	0.843	
SFA (%)	0.318	0.114		-0.231	0.277	0.397	0.045	*	0.057	0.793	
MUFA (%)	0.337	0.093		-0.024	0.910	0.270	0.183		0.002	0.994	
PUFA (%)	0.476	0.014	*	-0.316	0.133	0.222	0.276		-0.017	0.936	
NAS	0.211	0.301		-0.247	0.245	-0.069	0.737		-0.169	0.430	
**Physical activity**											
Total energy expenditure (Kcal)	-0.035	0.867		0.163	0.448	-0.091	0.660		0.039	0.856	

MT: muscle thickness, BM: body mass, EI: echo intensity, BMI: body mass index, baPWV: brachial-ankle pulse wave velocity, HDL-cholesterol: high-density lipoprotein cholesterol HOMA-IR: homeostatic model assessment for insulin resistance, QUICKI: quantitative insulin sensitivity check index, HOMA-β: homeostatic model assessment of beta cell function, SFA: saturated fatty acids, MUFA: monounsaturated fatty acids, PUFA: polyunsaturated fatty acids, NAS: nutritional adequacy score

* p <0.05

## Discussion

The present study examined factors associated with trunk MT/BM^1/3^ and EI in healthy young men and women in consideration of habitual dietary intake. The main results obtained were as follows: 1) in men, significant correlations were observed between trunk MT/BM^1/3^ and the percentages of energy from carbohydrates and PUFA and also between trunk EI and the percentage of energy from SFA, 2) in women, trunk EI significantly correlated with baPWV. These results suggest that factors associated with the amount of SM and content of intramuscular adipose tissue differ between young men and women. Since most previous studies did not examine the factors associated with the quantity or quality of SM it is difficult to determine the extent that each factor, including daily dietary nutrients, contributes to the trunk MT and EI. However, the effects of habitual dietary intake on the amount of SM and content of intramuscular adipose tissue in the trunk may be more prominent in young men than in young women.

In the present study, the index of the amount of trunk SM (trunk MT/BM^1/3^) significantly and positively correlated with the percentage of energy from PUFA and negatively correlated with carbohydrates in men (Table 2). PUFA is involved in muscle protein synthesis [33, 34]. An intervention study previously reported that cell size and the protein concentration of SM tissue in young and middle-aged healthy individuals were increased by PUFA supplementation because PUFA enhanced the anabolic response of muscle protein synthesis [35]. On the other hand, the intake of carbohydrates has been suggested to correlate with glucose demand and SM proteolysis [36]. A previous study demonstrated that a low-carbohydrate intervention inhibited muscle protein synthesis by increasing the production of ketone bodies and adrenaline [37]. Although the effects of PUFA and carbohydrates cannot be compared, each of these mechanisms may be mutually related to trunk MT in young men. In general, protein is one of the important nutrients that promotes muscle protein synthesis. However, in the present study, there was no significant correlation between trunk MT and daily protein intake (Table 2). A combination of protein intake and resistance exercise is essential to promote muscle protein content (e.g., [38, 39]). In this study, 30.7% of men and 29.1% of women had a protein intake below 13%, which is the minimum value recommended in the dietary reference intakes for Japanese (2020) [27]. In addition, none of the participants had an exercise habit. Taking these into account, inadequate protein intake and lack of exercise habit may be one of the reasons for no significant association between trunk MT and protein intake in the present study. On the other hand, the present study found no significant association between trunk MT and daily physical activity. The relationship between MT and physical activity is still controversial [40, 41]. Similar to the present study, Ikezoe et al. [40] reported that no correlation between daily physical activity and MT of the psoas muscles in elderly women. However, most previous studied involved elder people. Further studies are needed to elucidate the relationship between trunk MT and physical activity in young people.

Trunk EI, an index of non-contractile tissue, such as intramuscular adipose tissue, significantly and positively correlated with the percentage of energy from SFA in men (Table 2). The percentage of energy from SFA in our participants (7.6% for men and 8.7% for women) was higher than that of the tentative dietary goal in the dietary reference intakes for Japanese (2020) [27]. The intake of SFA has been suggested to relate with BM, total cholesterol, insulin resistance, and the inflammatory capacity of adipose tissue [42]. In addition, an intervention study previously demonstrated that a high intake of SFA increased the risk of abdominal obesity (waist circumference: >94 cm for men) in middle-aged individuals [43]. On the other hand, the content of intramuscular adipose tissue was shown significant relations with hyperlipidemia, insulin resistance, inflammatory response, and waist circumference [44, 45]. Although the mechanisms responsible for the relationship between SFA and trunk EI in young men have not yet been elucidated, lipid metabolism, insulin sensitivity, and inflammation may be involved.

The present study showed that habitual dietary intake significantly correlated with the indices of the amount of SM and content of intramuscular adipose tissue in young men only. Differences in lipid metabolism, blood profiles, and sex hormone secretion between the sexes may have contributed to this result. For example, in healthy middle-aged individuals, fatty acid synthesis in the liver is higher in men, whereas fatty acid oxidation is higher in women [46]. When fatty acid synthesis in the liver is accelerated, the amount of very low-density lipoprotein cholesterol, which is significantly related to the content of intramuscular adipose tissue, increases [47]. Very low-density and HDL-cholesterol, low-density lipoprotein-cholesterol, and triglyceride levels generally differ between young and middle-aged men and women [48]. In addition, the secretion of sex hormones is typically higher in young individuals than in older individuals [48]. However, SFA intake has been shown a significant relation with the content of intramuscular adipose tissue in middle-aged and older men and women [49]. Therefore, the relationship between trunk SM and dietary intake may differ depending on the age and sex of participants. Slater- Jefferies et al., [50] found that the phospholipid composition is more sensitive to dietary fat intake in female rats than in male rats. We showed that men were more sensitive to dietary fat intake than women, which supports their findings [50]. On the other hand, NAS, which assesses the intakes of 10 selected nutrients, did not significantly correlate with the indices of the amount of trunk SM or the content of intramuscular adipose tissue in both sexes (Table 2). Kitagawa et al. [14] reported a significant correlation between the content of intramuscular adipose tissue in the trunk and NAS in old men, but not in young men. NAS is evaluated by the intake of 10 nutrients (dietary fiber, vitamin B₆, vitamin D, vitamin K, folic acid, pantothenic acid, potassium, calcium, magnesium, and manganese) [14]. The present results imply that, at least in young healthy individuals, differences in lipid metabolism, blood profiles, and sex hormone secretion do not significantly affect the relationships between the 10 nutrients listed above and the amount of SM or content of intramuscular adipose tissue in the trunk. Since the relationship between the nutritional status and body composition may differ with age [51], further studies are warranted.

In women, habitual dietary intake did not significantly correlate with the amount of SM or content of intramuscular adipose tissue in the trunk. In terms of the amount of trunk SM, no variable showed a correlation following the exclusion of data from 1 participant with fasting blood glucose and insulin below the standard values recommended in Japan. Sato et al. [52] suggested that glucose tolerance and early-phase insulin secretion are impaired in young underweight (BMI: <18.5 kg/m^2^) Japanese women. In the present study, 33.3% of women were underweight. Therefore, the relationship between the amount of trunk SM and glucose metabolism may need to be examined in underweight and normal weight individuals in future studies. On the other hand, Ido et al. [9] reported that only abdominal MT, not other MT, was significantly associated with the metabolic risk scores in obese men and women aged 40 to 82. Tanaka et al. [53] also found that the quantitative ratio of trunk SM and visceral adipose tissue was significantly related to metabolic risk factors in middle-aged men. In contrast, the present study did not show a significant relationship between trunk MT and metabolic risk factors such as triglycerides, total cholesterol, HDL-cholesterol, fasting blood glucose, insulin, and HOMA-IR (Table 2). These discrepancies between studies may be due to the differences in physique, age, or sex of the subjects.

Trunk EI, an index of the content of intramuscular adipose tissue, correlated with baPWV (Table 2). Hasegawa et al. [54] reported that muscular lipids, such as extra- and intramyocellular lipids in the vastus lateralis muscle, positively correlated with baPWV in middle-aged and old individuals, but not in young individuals. The discrepancy between these findings and the present results may be attributed to a difference in the method used to evaluate ectopic fat and/or the targeted SM group. The mechanisms underlying the relationship between the content of intramuscular adipose tissue and arterial stiffness in young women have not yet to be elucidated [54]. However, inflammatory cytokines may be involved. Previous studies reported that the content of intramuscular adipose tissue was significantly associated with inflammatory cytokines, such as leptin, IL-6, and TNF-α [55]. These inflammatory cytokines have been suggested to correlate with the risk of arterial stiffness, endothelial dysfunction, insulin resistance, and diabetes [56, 57]. Further research on inflammatory cytokines is needed.

The present study has several limitations that need to be addressed. We used EI as an index of the content of intramuscular adipose tissue. However, EI reflects not only the infiltration of adipose tissue, but also other connective tissue and microvasculature, such as fibrosis and collagen [17]. In the present study, there was a group difference in the number of underweight, namely, 7.7% for men and 33.3% for women. This reflects the current situation in Japan, namely, a most young Japanese women experienced an epidemiologically relevant increase in underweight [58]. In addition, to avoid arbitrary selection, we did not select our subjects to adjust the proportion of underweight subjects between group. Although we adjusted MT with BM^1/3^, the influence of sex difference in physique may remain. The secretion of sex hormones is also related to group difference in trunk MT and EI; however, it is difficult to quantitatively assess its effects. In addition, our participants were all healthy young individuals. Since the present study had a cross-sectional design, a longitudinal study with larger sample sizes, including various physiques and health statuses, is needed in the future.

## Conclusion

The present study indicates that habitual dietary intake significantly correlates with the amount of trunk SM and the content of intramuscular adipose tissue in young men only. In women, arterial stiffness may be related to the content of intramuscular adipose tissue. Collectively, the results obtained herein suggest that factors associated with the amount of SM and content of intramuscular adipose tissue in the trunk differ between young men and women. These results imply that the trunk MT and EI have a potential as surrogate markers of habitual dietary intake in young men and arterial stiffness in young women.
